# Next Generation Sequencing Analysis in Patients Affected by Parkinson’s Disease and Correlation Between Genotype and Phenotype in Selected Clinical Cases

**DOI:** 10.3390/ijms26062397

**Published:** 2025-03-07

**Authors:** Andrea Pilotto, Mattia Carini, Roberto Bresciani, Eugenio Monti, Fabiana Ferrari, Maria Antonia De Francesco, Alessandro Padovani, Giorgio Biasiotto

**Affiliations:** 1 Neurology Unit, Department of Clinical and Experimental Sciences, University of Brescia, 25123 Brescia, Italy; 2 Neurology Unit, Department of Continuity of Care and Frailty, ASST Spedali Civili Brescia University Hospital, 25123 Brescia, Italy; 3 Laboratory of Digital Neurology and Biosensors, University of Brescia, 25123 Brescia, Italy; 4 Department of Molecular and Translational Medicine, University of Brescia, 25123 Brescia, Italy; 5 Highly Specialized Laboratory, ASST Spedali Civili of Brescia, 25123 Brescia, Italy; 6 Pediatrics, Mother’s and Baby’s Health Department, Poliambulanza Foundation Hospital Institute, 25124 Brescia, Italy; 7 Brain Health Center, University of Brescia, 25123 Brescia, Italy

**Keywords:** Parkinson’s disease, NGS, genetic background, clinical case

## Abstract

Parkinson’s Disease (PD) is the most frequent movement disorder and is second only to Alzheimer’s Disease as the most frequent neurodegenerative pathology. Early onset Parkinson’s disease (EOPD) is less common and may be characterized by genetic predisposition. NGS testing might be useful in the diagnostic assessment of these patients. A panel of eight genes (*SNCA*, *PRKN*, *PINK1*, *DJ1*, *LRRK2*, *FBXO7*, *GBA1* and *HFE*) was validated and used as a diagnostic tool. A total of 38 in sequence EOPD patients of the Parkinson’s Disease Unit of our Hospital Institution were tested. In addition, the number of the hexanucleotide repeats of the *C9ORF72* gene and the frequency of main HFE mutations were evaluated. Six patients were carriers of likely pathogenic mutations in heterozygosity in the analyzed genes, one of them presented mutations in association and another had a complex genetic background. Their clinical symptoms were correlated with their genotypes. In the cohort of patients, only the p.Cys282Tyr of *HFE* was significantly decreased in the dominant model and allele contrast comparison. Only one patient with one allele of *C9ORF72* containing 10 repeats was identified and clinically described. The clinical signs of sporadic and monogenic PD are often very similar; for this reason, it is fundamental to correlate genotypes and phenotypes, as we tried to describe here, to better classify PD patients with the aim to deepen our knowledge in the molecular mechanisms involved and collaborate in reaching a personalized management and treatment.

## 1. Introduction

Parkinson’s Disease (PD) is the most common movement disorder and the second most common neurodegenerative disorder after Alzheimer’s Disease [1]. The prevalence of PD is age related and it rises from a 2% in 60-year-old individuals to a 3% in the octogenarian population [2]. Early onset Parkinson’s disease (EOPD), on the other hand, is rare and accounts for about 10% of all diagnosed cases. It generally affects individuals between 40 and 60 years of age. The most common cause of EOPD is genetic [3]. This pathology constitutes a growing healthcare challenge, and the overall prevalence is believed to double in the next 20 years [4]. The most frequent symptoms of PD are resting tremors, bradykinesia, impaired posture and rigidity. In addition, there may be non-motor symptoms such as constipation, anosmia, cognitive impairment, sleep disorders and psychiatric symptoms [5,6,7]. The main pathological feature of PD is the loss of dopaminergic neurons in the substantia nigra caused by the accumulation of α-synuclein aggregates called Lewy bodies [8].

These aggregates characterize not only PD but also a series of overlapping diseases or synucleinopathies with peculiar clinical features [9].

PD is a multifactorial pathology that can be triggered by the interaction of both environmental and genetic risk factors. In the past twenty years, numerous families associated with PD have been tested to describe their genetic component. Consequently, several genes have been associated with PD, such as *LRRK2*, *SNCA* and *VPS35*, which are characterized by autosomal dominant inheritance. On the other hand, *PRKN*, *PINK1* and *DJ-1* are known for being associated with autosomal recessive EOPD, while others such as *ATP13A2*, *DNAJC6*, *FBXO7*, *PLA2G6* and *SYNJ1* determine atypical parkinsonism [3,10]. Generally, the clinical presentation of autosomal dominant PD is similar to that of patients affected by late onset idiopathic PD, and the most common variation reported is p.Gly2019Ser in *LRRK2* [11,12,13]. Instead, variations in genes such as *PRKN*, *PINK1* and *DJ-1* are responsible for early onset PD responsive to levodopa therapy and with a typical phenotype [3,10,14].

Variations in the *GBA1* gene do not cause Mendelian PD but are considered important risk factors [13,15]. This gene is responsible for the production of a housekeeping enzyme called beta-glucocerebrosidase involved in the degradation of glucocerebrosides in lysosomes. Although autosomal recessive Gaucher’s disease is strictly related to mutations in this gene, the carriers of heterozygous mutations have from a 2-fold to 20-fold higher probability to develop PD depending on the severity of the variation. Only one quarter of these carriers showed a family history of PD, while the rest of them were diagnosed as being affected by sporadic PD. Variations in this gene predispose to heterogeneous phenotypes ranging from asymptomatic carriers to forms similar to idiopathic PD or complex EOPD phenotypes with hypokinetic-rigid phenotype and major non-motor symptoms such as dementia/cognitive deficit, anosmia, dysautonomia and sleep disorders [3,14,16,17]. The analysis of the *GBA1* gene is a challenging diagnostic test because of the presence of the very similar pseudogene *GBAP1*, with 96% of homology and particular attention is needed in the choice of the analytical method [18].

PD is characterized by iron accumulation in the pars compacta of the substantia nigra but it is not known if the increased iron deposition is a trigger or a secondary event in the development of the disease [19]. Mutations in genes involved in iron metabolism have been associated with neurodegeneration in PD, such as the *HFE* gene, whose mutations are debated in the literature [20,21,22].

In our NGS panel we analyzed five genes (*LRRK2*, *DJ1*, *PINK1*, *PRKN* and *SNCA*) that are always included in the PD genetic panels identified by the NIH (National Institutes of Health) Genetic Test Registry. In addition to these, we analyzed the presence of mutations in the *GBA1* and *FBXO7* genes: the former is included as one of the major risk factors for PD while the latter is linked to an atypical form of parkinsonism [23]. Furthermore, we extended the analysis to the *HFE* gene to verify the presence of mutations linked to dysregulated iron metabolism in early onset PD. The non-coding expansion in the *C9ORF72* gene was also verified because of its hypothetical role as a predisposing factor for PD and other atypical parkinsonisms [24].

The purpose of the present study was, therefore, to analyze variations in these genes obtained from a cohort of 38 patients affected by familial PD and/or EOPD.

## 2. Results

### 2.1. Metrics

A total of 38 patients were tested with our expanded eight-gene panel containing *SNCA (PARK1/4)*, *PRKN (PARK2)*, *PINK1 (PARK6)*, *DJ1 (PARK7)*, *LRRK2 (PARK8)*, *FBXO7 (PARK15)*, *GBA1* and *HFE*. The metrics were calculated using the data of the first 26 patients analyzed in six NGS runs. The analysis generated an average (±SD) of 2,609,290 ± 903,175 bases for each patient. The mean ± SD of bases with Phred quality score (Qscore) ≥ Q20 was 2,460,439 ± 865,028 bases/patients. The mean of the reads produced for each patient was 13,988 ± 4802 (12,659 ± 5401 mapped reads, 89.86% ± 14.34% on target reads) with a mean length of 185.83 ± 2.48 bp. The mean coverage depth was 199.33X ± 83.68X. The chosen threshold for the variant calling was 20X coverage depth in agreement with the requirements for germline mutations [25,26]. All the regions below this threshold and all the pathogenic mutations were analyzed by Sanger sequencing. For each patient, all the covered regions were carefully analyzed with the IGV software (version 2.14.1) to verify the ion reporter variant call. In addition to NGS analyses, the number of hexanucleotide expansions in the *C9ORF72* gene were verified as reported below.

### 2.2. Validation Cohort

To validate the test, we analyzed some control samples with a confirmed clinical diagnosis of familial PD, one of which (25273) carried the pathogenetic mutation c.4321C>T p.Arg1441Cys (rs33939927) in heterozygous state in the *LRRK2* gene.

To check the specificity and the sensitivity of the method, we sequenced all the exons of the genes included in the panel in one patient (32163), with the dual purpose of setting up the PCRs to confirm the possible mutations by Sanger sequencing and to verify the accuracy of the NGS sequencing. In addition to this, in a second (32108) and third patient (32207) it was decided to sequence all exons of the *LRRK2* and *PRKN* genes, including non-coding regions covered by NGS sequencing. 

All the called variants obtained by NGS were confirmed by Sanger sequencing without identifying false positive or false negative results. Therefore, the specificity and the sensitivity of the NGS test after Sanger confirmation were equal to 100%. Considering the satisfactory results of the validation, the NGS panel was used for molecular diagnosis in the patients.

### 2.3. Identification of the Variants in the Prospective Cohort

A total of 38 patients with unknown genotypes and established clinical diagnosis of familial PD were analyzed. The mean number of variations found in each of the 38 patients was 68 (SD ± 8.97), with the largest number found in the *LRRK2* gene. We found 40 variations, mostly reported in the public databases, to have a MAF lower than 0.01. Thirty-one of these variantions were located in intronic or in untranslated regions apparently devoid of clinical significance or reported as benign/likely benign. Five mutations were found in the *GBA1* gene, as reported below. The remaining mutations identified were one in the *LRRK2* gene, two in the *PRKN* gene and one in the *SNCA* gene.

### 2.4. GBA1

The analysis of the *GBA1* gene is fundamental to distinguish it from the very similar pseudo gene *GBAP1*. The sequences of both the gene and pseudogene were compared, and primers were designed according to the difference within their base sequence in order to amplify specifically the exons and boundary regions of *GBA1*. The NGS analysis of the sequences of exons 9, 10 and 11, contained in the region with the most degree of homology between *GBA1* and *GBAP1*, was confirmed by Sanger sequencing for all the patients. As expected, the *GBA1* gene was found to express a wide number of pathogenic mutations in our cohort. We found five mutations in seven different patients (about 18.4% of the total) (Table 1). One of these patients was a compound heterozygous carrying the different mutations p.Thr408Met (rs75548401) and p.Leu483Pro (rs421016), while another patient carried the rare mutation p.Gly39Asp (rs200378040) which, in addition to the amino acid variation, could affect the splicing process changing the first base of exon 3.

### 2.5. Main Mutations of HFE Gene

All exons of the *HFE* gene were analyzed in the prospective cohort. We found the presence of the three main mutations associated with hemochromatosis and some frequent and harmless polymorphisms. The most frequent variation was p.His63Asp (H63D, rs1799945), which was found in seven patients: one patient expressed it in homozygosity, while the other six patients were heterozygous. The mutations p.Ser65Cys (S65C, rs1800730) and p.Cys282Tyr (C282Y, rs1800562) were found only in heterozygosity in two patients and one patient, respectively. The frequency of these mutations in the cohort of PD patients was compared with the frequency of the same mutations in the European (non-Finnish) population using data from the gnomAD database with a chi-square test. In the patient cohort, no difference of frequency was found when comparing the patients to the controls either by using the dominant model (DD+HD vs. HH, *p* = 0.1983) or allele contrast (D vs. H, *p* = 0.1566). Moreover, no significant difference was found by analyzing the p.Ser65Cys mutation (CC+CS vs. SS *p* = 0.4777; C vs. S, *p* = 0.4889). On the other hand, a significant difference was found when studying the mutation p.Cys282Tyr either using the dominant model or allele contrast (YY+CY vs. CC, *p* = 0.0477; Y vs. C *p* = 0.0495) (Table 2). In addition, although our population of patients was characterized by early onset PD with patients aged between 50 and 60 years, to enrich the analysis we compared the frequencies of the genotypes with a population of European healthy elderly people described by Willis et al. [27] which, however, was not completely suitable for comparison (mean age 88.3 years for men and mean of 91.8 women). In the comparison with this cohort of controls, the HFE mutations were all not significant. In particular, when the dominant model or allele contrast were used, the *p* values remained slightly above 10% (YY+CY vs. CC, *p* = 0.1128; Y vs. C *p* = 0.1115, but the number of people was much lower (1000 cases) than in the gnomAD database, and it may be that only by enlarging the cohort the statistical significance can be reached.

### 2.6. C9ORF72 Hexanucleotide Expansion

The GGGGCC hexanucleotide repeat in intron 1 of the *C9ORF72* gene was analyzed in the studied cohort of PD patients (Table 3). No difference in frequency was found when comparing the genotypes analyzed to those detected in the healthy population.

Homozygous genotypes with a low number of repeats were found in 16 patients. Among these, 10 patients showed two repeats, 2 patients displayed five repeats and 4 patients were characterized by eight repeats. A total of 22 patients showed heterozygous genotypes with different numbers of repeats between the two alleles. Among these patients, 11 were carriers of two and five repeats, 6 patients were characterized by two and eight repeats and 4 patients were heterozygous carriers with five and eight repeats. Only one patient had a heterozygous genotype composed of eight and ten repeats.

### 2.7. Private Mutations and Clinical Data

One female patient of Moroccan origin (34262) had the heterozygous mutation c.6055G>A, p.Gly2019Ser (rs34637584) of *LRRK2* gene. This mutation is known to be causative for PD with autosomal dominant segregation and is particularly frequent in the North African/Arab population (up to 39% in Berber Arab) [28]. She reported a family history for Alzheimer’s Disease (mother) and an unspecified walking disorder (father). The patient was characterized by an already advanced clinical phenotype (diagnosed in Morocco about 12 years before, when she was almost 40 years old) characterized by motor and non-motor symptoms, symmetrical mono-lateral tremor, significant bradykinesia, urinary incontinence, insomnia and anxiety–depression disorder.

The very rare heterozygous mutation c.310C>T, p.Arg104Trp (rs769099303) was found in the *PRKN* gene of the male patient 34203. This variation is reported in ClinVar to be of uncertain significance. In his medical history, he reported diabetes mellitus, systemic arterial hypertension, epilepsy in polytherapy and mycosis fungoides in phototherapeutic treatment. No family history for neurodegenerative diseases was reported. Clinically, the patient reported an initial tremor in the right upper limb when he was 56 years old, which then spread to the lower ipsilateral limb. From a motor point of view, he had a slow gait, mild generalized bradykinesia and no stiffness or reported falls. No non-motor symptoms were reported.

The compound heterozygous mutations p.Thr408Met (rs75548401) and p.Leu483Pro were found in the *GBA1* gene of another male patient (34310). He reported a family history for PD (paternal uncle). At 53 years of age he manifested a difficulty in the movement of the left leg associated with motor constraint of the upper contralateral limb. He presented a mild-to-moderate plastic hypertone in the upper limb. He also reported non-motor symptoms such as hyposmia, frequent nightly awakenings, vivid dreams and occasional sleep talking, but denied experiencing visual or auditory delusions.

**Figure 1:**
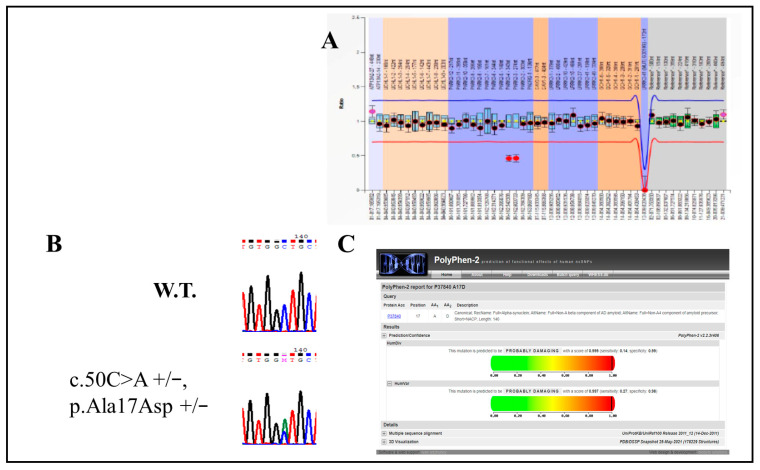
In this figure are represented: (**A**) the heterozygous deletion of exon 3 and exon 4 of the *PRKN* gene, (**B**) the new heterozygous mutation p.Ala17Asp in the *SNCA* gene (that the patient carried in association to the known mutation p.Asn409Ser in *GBA1* gene) and (**C**) the estimated pathogenicity of p.Ala17Asp using PolyPhen-2 software (Polymorphism Phenotyping, version 2) as an example of in silico simulation.

The female patient 28694 was the carrier of a more complex genetic background. This patient presented the heterozygous mutation p.Asn409Ser (rs76763715) in the *GBA1* gene in association with the novel heterozygous mutation p.Ala17Asp (no rs) in the *SNCA* gene. In addition, this patient was characterized by the heterozygous deletion of exon 3 and the initial part of exon 4 of the *PRKN* gene (Figure 1). Moreover, the patient carried the heterozygous mutation p.His63Asp (rs1799945) of the *HFE* gene. The patient reported a positive family history for Alzheimer’s Disease (father), an unspecified cognitive decline (mother) and Lewy body disease (paternal aunt) but no other diseases worthy of note in the anamnesis. She became symptomatic at the age of 43 and she reported a progressive motor impairment in the right hand, bradykinesia and mixed tremors in the upper limbs, moderate bradykinesia in the lower right limb and plastic-spastic hypertone in the upper right limb. She reported a preserved sleep-wake rhythm, urge urinary incontinence episodes and a severe anxiety–depressive syndrome in treatment with clozapine in off-label use.

The male patient 32976 carried the rare p.Gly39Asp (rs200378040) mutation in the *GBA1* gene. This mutation is not reported in the ClinVar database and is very rare in the gnomAD database, where it is reported only in heterozygosity with an 8.0 × 10^−6^ value of allele frequency. This patient had a family history for PD (mother) with a non-tremor dominant phenotype. At 57 years of age he manifested bilateral resting tremors and mild plastic hypertone to the upper limbs. In addition, he manifested bradykinesia, camptocormic gait and slight hypomimia. He reported a preserved sleep-wake rhythm and sleep talking but no involuntary movements. He complained of irregular constipation but had no urinary incontinence, orthostatic hypotension or cognitive–behavioral disorders except for worsening anxiety.

The female patient 19GM1966 was characterized by the heterozygous mutation p.Ala82Glu (rs55774500) in the *PRKN* gene. The patient had a history of buccal dystonia and dyskinesia of the tongue during speech with dysarthria that started when she was 49 years old. These symptoms were reported to be absent at rest, did not present fluctuations throughout the day and were not associated with dysphagia. She did not report a family history for neurological diseases and denied major pathologies.

Lastly, the male patient 33830 had a family history of cognitive decline (grandmother). He had no history of extrapyramidal diseases. He reported an anamnestic car accident at a young age but denied head traumas. He was characterized by a resting tremor in the left hand that appeared when he was 45 years old, associated with mild plastic rigidity and a tendency to micrographia. He did not report any non-motor symptoms except for a tendency to orthostatic hypotension. No significant mutations were found in the NGS sequencing, but a heterozygous genotype with eight to ten repetitions of the *C9ORF72* gene was found (the correlation between genotypes and phenotypes is summarized in Table 4).

## 3. Discussion

Parkinson’s Disease is a pathology characterized by a complex etiology. The contribution of the genetic background still remains elusive and explains only a minority of the new diagnosed cases. The study of the genetic component in PD patients may be particularly important in familial cases and in those patients presenting an early-onset disease [29]. The NGS technology is especially suitable to test patients affected by pathologies with a complex genetic architecture.

### 3.1. Choice of the Genes

In recent years, numerous NGS panels have been used to study PD, and they can vary from small panels containing five or six genes to others including up to a hundred genes. This wide range of panels combined with the limited knowledge of the correlation between genetic mutations and the clinical phenotype can restrain the physicians from prescribing or selecting the correct genetic test [30]. Although there are no precise guidelines on what panel to select in the diagnosis of PD, at the moment, some groups of experts have proposed algorithms that take into account family history, ethnicity and clinical signs [31,32].

A recent work analyzed 11 commercial CLIA-certified (Clinical Laboratory Improvement Amendments) PD genetic panels selected in a database of orderable tests supported by the NIH (National Institutes of Health). All these panels comprised at least five genes (*LRRK2*, *PARK7*, *PINK1*, *PRKN* and *SNCA*), and the largest of them included sixty-two genes [23]. Considering that large panels can generate a great number of VUS (variant of uncertain significance), which might complicate the genetic counseling, we chose to limit our panel to the five genes mentioned above, with the addition of three other genes: *GBA1*, *FBXO7* and *HFE*. *GBA1* and *FBXO7* were chosen because of their role in PD [3,15] and atypical parkinsonism, while *HFE* was included because of its correlation to brain iron disorder, which we have been studying for years [21,22,33].

### 3.2. Comment on the Mutations and Clinical Data

Among the causative mutations of the *LRRK2* gene, the most common is p.Gly2019Ser. This was present in one patient of Moroccan origin, who was characterized by an already advanced clinical phenotype (that had started about 12 years before) characterized by motor and non-motor symptoms, symmetrical monolateral tremor, significant bradykinesia, urinary incontinence, insomnia and mood tone deflection associated with anxiety. This mutation is particularly present in the Maghrebi population, where it represents a genetic cause of familial PD in at least 40% of the patients [28,34] and it is responsible for a clinical phenotype very similar to that found in patients affected by idiopathic PD [12].

The very rare heterozygous mutation p.Arg104Trp (rs769099303) of the *PRKN* gene was found in patient 34203. The clinical picture of this patient was characterized by a predominantly hypokinetic phenotype, presenting symmetrical tremors and symptoms of non-motor type. The phenotype appeared to correspond with that reported by Rajan et al. [3] in association with *PRKN* mutations. This mutation substituted the positively charged Arginine for a hydrophobic Tryptophan. This amino acid variation of the parkin sequence had already been found with a very low MAF (2.9 × 10^−5^), but its pathological contribution to the clinical phenotype remains unclear. The in silico prediction of the effect of this mutation was ‘likely pathogenic’, with a score of 4 out of 6 (Table 5) [35]. In detail, Arg104 belongs to an unstructured 66-amino acid linker that connects the UBL N-terminal domain to a hydrophobic groove of the RING0 domain in the inactive form of parkin. This linker contains different amino acids from Gln 100 to Ser108, but especially Leu102, Val105 and Leu107 are essential in this connection.

The role of Arg104 is crucial when parkin changes to its activated form: by binding to the negative residue Asp60 contained in the UBL N-terminal domain it can form a stable salt bridge and thus prevent the interaction between the UBL and the RING0 domains. In phylogenesis, the length of the linker is conserved at least until the zebrafish (*Danio rerio*) on the evolution scale. In particular, Arg104 is highly conserved as part of the region comprising Ser101 and Leu123, and is characterized by the highest degree of preservation in the central part of the linker sequence [36,37]. The substitution of the Arg104, which is replaced by a Trp, could prevent the formation of the salt bridge, destabilizing the active form of parkin, decreasing its functionality and contributing to generate a pathological condition.

Patient 34310 carried two heterozygous mutations in the *GBA1* gene, p.Thr408Met (rs75548401) and p.Leu483Pro (rs421016). Both variations are known to be risk factors for PD, and the latter is one of the two most frequent GBA mutations (the other is p.Asn409Ser). The clinical phenotype may be compatible with the phenotypic characteristics of *GBA1* mutations reported in the literature [3,13]. The effect of these known mutations is an alteration of the protein functionality, compromising both the activity of the enzyme site and protein folding [3,14].

Patient 28694 had a very complex genetic background. In particular, she carried the p.Asn409Ser (rs76763715) heterozygous mutation in the *GBA1* gene in association with a new p.Ala17Asp (no rs) heterozygous mutation in the *SNCA* gene. In addition, this patient was characterized by the heterozygous deletion of exon 3 and the initial part of exon 4 of the *PRKN* gene. Her clinical phenotype was not simple to classify: the onset symptom was a motor constraint on the right hand, but then she developed severe bradykinesia, mixed tremor in the upper limbs and plastic-spastic rigidity. Among the non-motor symptoms, instead, she was characterized by a severe mixed anxiety–depressive disorder. The hypokinetic/rigid picture seems to be in line with what is reported in the literature about *PRKN* and *GBA1* mutations, while the important anxiety–depressive syndrome follows the non-motor symptomatology often associated with *GBA1* [3,13]. The new mutation p.Ala17Asp in the *SNCA* gene cannot be traced either in the literature or in the most common databases, but the in silico prediction of its pathogenicity allows us to estimate it as a ’probably damaging’ mutation, with a score of 6 out of 6. Like the most well-known *SNCA* mutations, such as p.Ala30Gly and p.Ala30Pro, the new mutation p.Ala17Asp alters the N-domain in the α-synuclein protein terminal, replacing a non-polar amino acid such as Alanine with a negatively charged Aspartate. This mutation could likely alter the interaction and contact between N-domains and C-terminals of the protein [29]. In addition, we could speculate that the mutation of glycosylceramide beta 1 and α-synulein simultaneously present in this patient might influence each other. Functional studies described an interplay between these two proteins, which showed an inverse correlation. The mutant forms of *GBA1* augment the level of α-synulein, and this protein could inhibit the GBA functionality [38]. Moreover, the α-synulein mutants p.Ala53Thr and p.Ala30Pro seem to induce mitochondrial fragmentation, and α-synulein could interfere with PINK1/parkin signaling, influencing the mitochondrial fission [39]. Although the α-synulein effects on the mechanisms of mitochondrial proteins remain to be fully clarified, a possible role of these pathological interactions could not be excluded in this patient.

The mutation p.Gly39Asp (rs200378040) in the *GBA1* gene was found in heterozygosity in a 63-year-old male patient, but this mutation is not reported in the ClinVar database and has been found only thirteen fold among 1,613,334 alleles once in an African/African American male and twelve fold in European population (non-Finnish, gnomAD). The patient’s phenotype corresponded to what is mentioned in the literature for *GBA1* mutations, especially regarding the psychiatric symptoms and REM sleep disorders. When taking into account the possible molecular effect, the substitution of the small and neutral amino acid Glycine with a negatively charged Aspartic acid could be hypothesized as remarkable, but the in silico prediction resulted to be ‘disease causing’, with a score of 1 out of 6, and might be tolerated or neutral. This variation falls in the last amino acid of the *GBA1* signal peptide, which may be constituted by 39 or 19 amino acids depending on the position of the start codon, which determines the transcribed isoform [40]. Usually, the cleavage site of the signal peptide in mammals is constituted by three hydrophobic amino acids from position −3 to −1 before the first residue of the mature protein, and the variation from Glycine to Aspartic might interfere with the cut made by the peptidase SPase [41]. In addition to this, the variation c.116G>A falls on the first base of exon 3 and could interfere with the splicing process of mRNA causing the loss of the acceptor site as predicted by the software Mutation Taster (version 2021) [42]. This software is the only solution used for the in silico simulation that reproduces the possible effect on a splicing site. Interestingly, this software classifies the mutation site as ‘disease causing’.

The patient 19GM1966 had only one mutation p.Ala82Glu (rs55774500) in the *PRKN* gene. This mutation is reported in ClinVar in association with autosomal recessive juvenile onset PD but is referred to as ’likely benign’. However, the patient’s clinical phenotype was peculiar, characterized by buccal dystonia, dyskinesia of the tongue during speech resulting in dysarthria. Unfortunately, we lost track of the patient without ever being able to monitor the possible evolution of the symptoms or reach a definitive diagnosis. As seen in patient 34203, this variation affects the unstructured 66-amino acid linker linking the N-terminal of the UBL domain to a hydrophobic groove of the RING0 domain in the inactive form of parkin. Replacing a non-polar amino acid such as Alanine at position 82 with a negatively charged Glutamic acid could interfere with the interaction between the UBL and RING0 domains, destabilizing the active form of parkin [36,37]. However, the clinical picture is in line with what was stated by Millar Vernetti et al. [43] and Rajan et al. [3]. These authors reported how the phenotypic spectrum of patients with variations in *PRKN* can be highly variable and how sometimes it may start with isolated dystonia, although especially in the lower limbs.

### 3.3. HFE Mutations

All exons of the *HFE* gene were analyzed in the prospective cohort. We found the presence of the three main mutations associated with hemochromatosis. The most numerous among those variations was p.His63Asp (H63D, rs1799945), which was found in homozygosity in one patient, while six patients presented it in heterozygosity. The mutations p.Ser65Cys (S65C, rs1800730) and p.Cys282Tyr (C282Y, rs1800562) were found only in heterozygosity in two patients and one patient, respectively. The frequency of these mutations in the cohort of the PD patients was compared with the frequency of the same mutations in the European (non-Finnish) population of the gnomAD database with a chi-square test. The frequencies of all the genotypes were analyzed, and, in addition, the dominant model and allele contrast were used. Studying the p.His63Asp mutation in the general comparison (*p* = 0.3892), in the dominant model (DD+HD vs. HH, *p* = 0.1983) or in the allele contrast (D vs. H, *p* = 0.1566), no difference in frequency was found. The same results were obtained analyzing the p.Ser65Cys mutation (*p* = 0.7666; CC+CS vs. SS *p* = 0.4777; C vs. S, *p* = 0.4889).

When analyzing the p.Cys282Tyr mutation, no significant difference was found in the general comparison (*p* = 0.1402), while a significant decrease of the mutation was found using both the dominant model and the allele contrast (YY+CY vs. CC, *p* = 0.0477; Y vs. C *p* = 0.0495) (Table 2).

The analysis of the frequencies of p.His63Asp and p.Ser65Cys mutations in our cohort of patients did not show evidence of association with risk of PD when compared to the European (non-Finnish) population of the gnomAD database. We found a decrease in the frequency of the allele coding Y of the mutation p.Cys282Tyr when we used both the dominant model and allele contrast (*p* = 0.0447 and *p* = 0.0495, respectively). The same finding was reported in a previous paper of ours where we studied *HFE* mutations, including the p.Cys282Tyr, in a more numerous PD cohort [21]. Furthermore, when the comparison was performed using a European healthy elderly population of about one thousand people but not perfectly matched for age with our cohort of patients characterized by early onset (50–60 years old), the *p* values remained around 10%. However, the number of patients analyzed was too small, and this figure must be verified by enlarging both the number of patients and the cohort of matched controls. As mentioned above, the role of these mutations is not yet clear in the literature, and in particular the p.Ser65Cys mutation had only a marginal role in the studies of p.His63Asp, probably because of its low frequency [44,45].

The p.His63Asp mutation is a common polymorphism, especially in the European population, and, therefore, is also well represented in the PD population. Although the p.His63Asp effect can be expected to increase cellular iron accumulation, which may worsen oxidative stress and α-synuclein aggregation in PD patients, no association between this mutation and the increase of the risk to develop the pathology has been described. In contrast, the molecular evidence obtained studying the cellular pathways involved in PD pathology showed that the mutation of p.His63Asp had a protective role in both cellular and animal models by improving ROS defenses and decreasing the toxic effects of protein aggregation [46,47,48]. Therefore, *HFE* polymorphisms may be important to explain the heterogeneity of the clinical manifestation of patients affected by PD, which may include both motor and non-motor symptoms. In particular, the diminution of the striatal binding ratio could be a peculiar characteristic of p.His63Asp carriers among PD patients, contributing to the variation of the clinical presentation. This characteristic might confer importance to the genotyping of *HFE,* with the dual benefit of understanding the heterogeneous clinical presentation and evaluating possible therapeutic strategies regarding the field of iron metabolism [20,49].

### 3.4. General Analyses C9ORF72

The role of the expansion of the hexanucleotide GGGGCC of the *C9ORF72* gene was analyzed in PD. The studies reported in the literature applied a variable threshold from 30 to 60 repeats to prove the pathogenic effect. Two studies reported that the frequency of this expansion in PD patients was less than 1% in the Caucasian population [50,51], while other studies did not find carriers of the *C9ORF72* expansion, especially in European and North American patients [52,53,54,55,56,57,58,59]. In addition to this, Asian patients do not seem to carry the *C9ORF72* expansion [60,61].

A meta-analysis regarding more than 7000 patients of all ethnic groups was conducted by Genetic Epidemiology of Parkinson’s disease (Geo-PD), but this study failed to achieve a statistical significance when comparing patients to controls when the threshold was shortened to ≥17 hexanucleotide repeats [62]. Nuytemans et al. found that 2% of the patients were characterized by an intermediate expansion (21 to >30 hexanucleotide repeats) and reported that the statistical analysis seemed to suggest that an expansion in this range might be a risk factor for PD. However, the interval of confidence was very large, probably due to the small number of intermediate expansion carriers [55]. An association with presence of intermediate expansion was found in a study of the Chinese population, but in this case a shorter cut-off of >7 repeats was used [63]. The very large study performed by Geo-PD tried to verify the contribution of small repeat expansions to the susceptibility of the PD and they hypothesized a slight increase of risk for disease susceptibility when the number of repeats increased. When taking size effect into account, a statistical significance would not be reached for a number of repeats greater than 9 or 10 [62]. All these considerations corroborate the fact that the definition of intermediate number of repeats in *C9ORF72* is still not so clear at the moment [64]. However, some authors indicate that an intermediate number of repeats between 10 to 20 may be a risk factor for PD [55,62,63]. In addition, some observations seem to suggest that a smaller or intermediate number of repeats could be more linked to the susceptibility of parkinsonism rather than predispose to PD [65]. To sum up this part, the large *C9ORF72* expansions do not seem to play a major role in typical PD. The difficulty of classifying the complex phenotypic presentation, especially in syndromes with Parkinsonian clinical signs, makes it difficult to standardize the cohorts of patients. For this reason, the comparison among them and the general picture remains unclear. Well standardized studies would be needed also for atypical parkinsonism to define the possible role of *C9ORF72* expansions and the role of the intermediate number of repeats. Finally, the described somatic instability of the repeat number with larger count in brain compared to peripheral blood contributes to prevent a good correlation between genotype and phenotype [65].

In our cohort of patients, we tested the number of hexanucleotide repeats of the *C9ORF72* gene and we found a clear prevalence of standard genotypes. The genotypes of the patients and their frequencies were comparable with those reported for the healthy population, as expected, even considering the small number of patients enrolled in our cohort. Only one patient had a heterozygous genotype characterized by 8–10 repeats. This patient was negative for mutations in the genes analyzed by the NGS panel, and he had a clinical phenotype characterized by left hand tremor at rest associated with mild plastic hypertonia and tendency to orthostatic hypotension. These symptoms could agree with the description of Bourinaris and Houlden [65], but all the considerations reported before underline the fact that reaching a consensus on the threshold of repetitions in *C9ORF72* is not yet possible [24,64].

### 3.5. Importance of Defining a Phenotype with a Genotype

In conclusion, recent studies have shown a more significant role of genetics in PD than previously believed, revealing a contribution of gene mutations with limited penetrance in sporadic PD [9].

The clinical manifestations in the sporadic and the monogenic form of PD are often very similar. The tracking of the correlation between the clinical phenotype and the mutation in the genes involved in PD could be very useful to personalize the management and the treatment of patients. However, the evolution of the pathology can differ greatly from individual to individual, and the understanding of the molecular causes of these dissimilar evolutions may allow us to find a new therapeutic approach to redefine the current therapeutic protocols. In addition, the correlation between genotype and phenotype needs to be clarified further, since some genes can be responsible for different pathologies, such as the *LRRK2* mutation, which is implicated in PD, Lewy Bodies Disease or Multiple System Atrophy. The role of this mutation in modifying genes needs to be more deeply researched [29,66,67]. Much work is still needed in this field, and it is fundamental to continue studying the genetic risk factors at a biological and molecular level so that it will become possible to classify the PD patients in groups with a better-defined genotype–phenotype correlation. Only a combination of the new genetic evidence and the already known pathophysiological mechanism might lead to a better understanding of the PD evolution and to the identification of new target therapies [68].

## 4. Material and Methods

### 4.1. DNA Extraction

Genomic DNA has been extracted starting from 400 µL of EDTA-added peripheral blood using the kit Maxwell^®^16 Blood DNA Purification System (Promega, Madison, WI, USA). DNA concentration has been quantified using Qubit 3.0 Fluorometer (Thermo Fisher Scientific) and the Qubit dsDNA HS Assay Kit (Thermo Fisher Scientific). The final concentration was usually 20/40 ng/µL.

### 4.2. NGS Sequencing

We used the software AmpliSeq Designer v7.8.7 (Thermo Fisher Scientific, Waltham, MA, USA) to design a panel of primers to amplify and to sequence 8 genes: *SNCA (PARK1/4)* (NM_000345.4), *PRKN (PARK2)* (NM_004562.3), *PINK1 (PARK6)* (NM_032409.3), *DJ1 (PARK7)* (NM_007262.4), *LRRK2 (PARK8)* (NM_198578.4), *FBXO7 (PARK15)* (NM_012179.3), *GBA1* (NM_000157.4) and *HFE* (NM_000410.4). This panel of primers was designed to cover all the codifying exons and at least 10 intron bases flanking every exon. However, the panel covered the 5′UTR and the 3′ UTR regions of the coding mRNAs and the regions codifying the protein isoforms. The target region of the panel corresponded to 48.37 Kb. The estimated coverage in silico was 95.5 percent of the region targeted, and it meant that 1749 bases were not covered. The primers were divided into two pools, both generating 134 amplicons. The length of the fragments was between 125 and 375 bases. The libraries for each patient were amplified by two separate reactions, one for each pool, and were labeled with the same molecular barcode, specific for each patient (Ion Xpress Barcode Adapters 1-16 Kit, Thermo Fisher Scientific, Waltham, MA, USA). The libraries were purified, amplified and again purified by AMPure XP reagent (Beckman Coulter, Brea, CA, USA) applying the manufacturer’s instructions. After DNA quantification of the barcoded libraries using the Qubit dsDNA HS Assay Kit (Thermo Fisher Scientific, Waltham, MA, USA), they were diluted in equimolar concentration, pooled and then amplified in an emulsion PCR using the Ion PGMTemplate OT2 200 Kit with a Ion One Touch 2 Instrument (Thermo Fisher Scientific, Waltham, MA, USA). An Ion OneTouch ES Instrument (Thermo Fisher Scientific, Waltham, MA, USA) was used to concentrate Ion Sphere Particles (ISPs). The quality control of the enrichment steps was verified with the Ion Sphere Quality Control Assay (Thermo Fisher Scientific, Waltham, MA, USA) using a Qubit 2.0 Fluorometer. The sequencing of the obtained ISPs was performed using an Ion Torrent PGM System (Thermo Fisher Scientific, Waltham, MA, USA) with the Ion PGM Hi-Q Sequencing Kit (Thermo Fisher Scientific, Waltham, MA, USA), charging Ion 314 chips and using 500-flow runs.

### 4.3. NGS Data Analysis

Alignment to the hg19 human reference genome (GRCh37) and variant calling were made in the Ion Torrent Server (Thermo Fisher Scientific, Waltham, MA, USA) using the Torrent Suite Software version 5.12.2 (Thermo Fisher Scientific, Waltham, MA, USA) and applying the VariantCaller plugin (Thermo Fisher Scientific, Waltham, MA, USA). Ion Reporter 5.0 Software (Thermo Fisher Scientific, Waltham, MA, USA) was used to perform variant analysis identifying single nucleotide variations (SNV), insertions or deletions (indels). All sequences and the called variations were analyzed using the Integrative Genomic Viewer (IGV software (version 2.14.1), Broad Institute, University of California, USA) to verify the presence of false positives or false negatives in the variant calling. Annotation of the variants was made using the convention proposed by the Human Genome Variation Society [69]. The impact of the variations identified and the Minor Allele Frequency (MAF) were evaluated and assigned by consulting the following databases: LOVD, ClinVar and gnomAD [70,71,72]. A sample was loaded in duplicate in the same run as intra-assay control, and the same sample was analyzed in three consecutive runs as inter-assay control. No difference among the variations detected was found.

### 4.4. Sanger Sequencing

The patients of the validation cohort were partially sequenced by Sanger sequencing. All patients with variations found by NGS sequencing and belonging to both the validation and prospective cohorts were verified and confirmed by Sanger sequencing. One patient was entirely sequenced by Sanger sequencing to confirm the variations found by Ion PGM sequencing. In addition, the genes *LRRK2* and *PRKN* were entirely sequenced in two further patients to confirm the variations found by both the sequencing techniques. Considering the complete overlap between the results obtained using Sanger sequencing and Ion PGM sequencing, we decided to check only the variations causing amino acid change in the protein sequence or known to be pathogenic.

### 4.5. MLPA Analysis

The deletions or duplications of exons of the studied genes were analyzed by multiple ligation-dependent probe amplification (MLPA) using SALSA MLPA probemix P051-D1 and a P-052-D1 kit as described by the protocol of the manufacturer and analyzed with the Coffalyser software v.220513.1739 (MRC-Holland, Amsterdam, The Netherlands).

### 4.6. GBA1 Gene Analysis

In order to analyze specifically the *GBA1* gene and discriminate it from the pseudogene GBAP1, we designed a specific pool of primers (Carlo Erba Reagents, Milan, Italy) based on the difference of the DNA bases between the sequences of the gene and the pseudogene. The primers were designed to analyze the whole exon and the flanking regions. The amplification was made in 25 μL of final volume containing 50 ng of genomic DNA, 10 mM Tris-HCl (pH 8.3), 50 mM KCl, 2mM MgCl_2_, 200 μM of each dNTPs, 0.75 U of AmpliTaq Gold (Thermo Fisher Scientific, Waltham, MA, USA) and 12.5 pmol of each specific primer. Appropriate conditions for amplification of the PCR fragments are shown in the Supplemental Materials (Supplementary Table S1).

### 4.7. Analysis of Hexanucleotide Expansion of C9ORF72

The hexanucleotide expansion analysis of the *C9ORF72* gene is a three-step test to allow for the exact dimensioning of expansions of intermediate length as well as the identification of large expansions: the two first steps are based on the polymerase chain reaction (PCR) method, namely a genotyping PCR and a 5′ repeat-primed (RP) PCR, followed by an analysis of the length of fragments of both products by agarose gel electrophoresis (for genotyping) and capillary fluorescence electrophoresis. RP-PCR is performed in a 25 μL reaction, containing 10 ng of DNA, 1.5 mM of MgCl2, 10% dimethyl sulfoxide (DMSO), 0.25 mM of dATP, dCTP, dTTP plus 0.25 mM of 7-deaza-dGTP, 1 μM of forward-labelled primer FAM, 0.5 μM of reverse primer and 1 μM of anchor-tailed primer reverse, with the following protocol: 95 °C 10 min, 5 cycles (30 s 94 °C, 2 min 62 °C, 3 min 72 °C), 15 cycles (30 s 94 °C, 2 min 60 °C, 3 min 72 °C), 25 cycles (30 s 94 °C, 2 min 58 °C, 3 min 72 °C) and final extension (10 min 72 °C) [73].

### 4.8. Prediction of the Effect of Mutations

The assignment of the pathogenicity for the known mutations was based on what had been reported in the reference databases ClinVar and gnomAD [71,72]. The new variations with uncertain significance (VUS) were analyzed by using 6 different software solutions for computational prediction of pathogenicity [42,74,75,76,77,78]. When we assessed the various results, each mutation was assigned a score from 1 to 6 constructed by summing up the number of pathogenic predictions on the total of the 6 simulations carried out.

### 4.9. Statistical Methods

Considering the small number of patients analyzed, in addition to general comparison, only two models were used to compare the alleles for the *HFE* mutations p.His63Asp, p.Ser65Cys and p.Cis282Tyr: dominant model and allele contrast. The comparison of the distribution of the genotypes between the studied cohort and the control database was performed using a χ^2^ test.

## 5. Patients

The patients enrolled in this study were assessed for suspected EOPD by the Neurologists of the Parkinson’s Disease Unit of the 2nd Neurology Department of ASST Spedali Civili of Brescia. The prospective cohort comprised 38 patients (24 males and 14 females) diagnosed between June 2018 and June 2021. The informed consent was collected for each patient before the beginning of the genetic analysis. As a validation cohort, we selected 4 patients submitted for the conventional genetic test for the same pathology.

## Figures and Tables

**Table 1:** *GBA1* Mutation.

Patient Code	*GBA1* NM_000157.4	Protein	Zigousity	rs	ClinVar
32976	c.116G>A	p.Gly39Asp	+/−	rs200378040	Not available
34267	c.1226A>G	p.Asn409Ser	+/−	rs76763715	Pathogenic/Likely pathogenic Risk factor
34310	c.1223C>T	p.Thr408Met	+/−	rs75548401	Likely benign Uncertain significance
	c.1448T>C	p.Leu483Pro	+/−	rs421016	Pathogenic Risk factor
35118	c.1223C>T	p.Thr408Met	+/−	rs75548401	Likely benign Uncertain significance
28694	c.1226A>G	p.Asn409Ser	+/−	rs76763715	Pathogenic/Likely pathogenic Risk factor
33231	c.1093G>A	p.Glu365Lys	+/−	rs2230288	Likely benign, Uncertain significance Risk factor
32931	c.1448T>C	p.Leu483Pro	+/−	rs421016	Pathogenic Risk factor

Table 1: In the table are reported the patient code, the change of the cDNA bases and the effect in protein sequence, the rs reported in dbSNP database and the clinical effect reported in ClinVar.

**Table 2:** Genotypes and allele frequencies of the HFE gene compared with the European (non-Finnish) population reported in the gnomAD database.

Mutation	+/+	+/−	−/−	*p* Value	Dominant Model		*p* Value	Allele Contrast		*p* Value
H63D	DD	HD	HH		DD+HD	HH		D	H	
Patients	1	6	31	0.3892	7	31	0.1983	7	69	0.1566
Controls	13363	150441	426128		163804	426128		177167	1002697	
S65C	CC	CS	SS		CC+CS	SS		C	S	
Patients	0	2	36	0.7666	2	36	0.4777	2	74	0.4889
Controls	159	18883	570962		19042	570962		19201	1160807	
C282Y	YY	YC	CC		YY+YC	CC		Y	C	
Patients	0	1	37	0.1402	1	37	**0.0477**	1	75	**0.0495**
Controls	3173	77461	509366		80634	509366		83807	1096193	

Table 2: *p* values were calculated using a χ^2^ test.

**Table 3:** *C9ORF72* genotypes.

Genotype	2-2	5-5	8-8	2-5	2-8	5-8	8-10
N. Patients	10	2	4	11	6	4	1

**Table 4:** Genotype–phenotype presentation.

Patient Code	Gene	Mutation	Familial History	Clinical Phenotype
34262	*LRRK2*	p.Gly2019Ser	yes	Symmetrical mono-lateral tremor, significant bradykinesia, urinary incontinence, insomnia and anxiety-depression disorder
34203	*PRKN*	p.Arg104Trp	no	Initial tremor in the right upper limb and then spread to the lower ipsilateral limb, slow gait and mild generalized bradykinesia
34310	*GBA1*	p.Thr408Met p.Leu483Pro	yes	Difficulty in the movement of the left leg associated with motor constraint of the upper contralateral limb, mild-to-moderate plastic hypertone in the upper limb, hyposmia, frequent nightly awakenings, vivid dreams and occasional sleep talking
28694	*GBA1* *SNCA* *PRKN * *HFE*	p.Asn409Ser p.Ala17Asp del ex3–4 p.His63Asp	yes	Progressive motor impairment in the right hand, bradykinesia and mixed tremor in the upper limbs, moderate bradykinesia in the lower right limb and plastic-spastic hypertone in the upper right limb, urge urinary incontinence episodes and a severe anxiety-depressive syndrome
32976	*GBA1*	p.Gly39Asp	yes	Bilateral resting tremor and mild plastic hypertone to the upper limbs, sleep talking, irregular constipation and worsening anxiety
19GM1966	*PRKN*	p.Ala82Glu	no	Buccal dystonia and dyskinesia of the tongue during speech with dysarthria
33830	*C9ORF72*	8-10 x GGGGCC	yes	Resting tremor in the left hand, mild plastic rigidity and a tendency to micrographia and tendency to orthostatic hypotension

**Table 5:** In silico prediction of the effects of new/rare mutations.

Gene	Mutation	Polyphen2	Mutation Taster	Provean	SIFT	SNAP2	SNPs&GO	Prediction
*GBA1*	p.Ala39Asp	Benign	Disease causing	Neutral	Tolerated	Neutral	Neutral	1 out of 6
*PRKN*	p.Arg104Trp	Probably damaging	Polymorphism	Deleterious	Damaging	Effect	Neutral	4 out of 6
*SNCA*	p.Ala17Asp	Probably damaging	Disease causing	Deleterious	Damaging	Effect	Disease	6 out of 6

## Data Availability

Data are contained within the article and Supplementary Materials.

## Supplementary Materials

The following supporting information can be downloaded at: https://www.mdpi.com/article/10.3390/ijms26062397/s1.
