# Supplementary material for: Next Generation Sequencing Analysis in Patients Affected by Parkinson’s Disease and Correlation Between Genotype and Phenotype in Selected Clinical Cases
Source: Int J Mol Sci. 2025 Mar 7;26(6):2397. doi: 10.3390/ijms26062397 (PMC11942189; doi:10.3390/ijms26062397)
Supplement: Supplementary file 1 [file ijms-26-02397-s001.zip › ijms-3479531-supplementary.pdf]

Supplementary Table S1. Primers *GBA1*.

Exon	Primers Sequence	Annealing Temperature	Fragment Lenght bp
1	1F GCCgGAATTACTTGCAGGgc<br>1R TTTGGGTGCCCATGGCCc	58 °C	456
2	2F GCAGCTAAGCCCTGCCCa<br>2R ggcaacagagtaagactctg	58 °C	359
3	3F ACCGTgTTCAGTCTCTCCtAg<br>3R ataagggTATCAGTaCCCAGc	58 °C	381
4	4F gCTGGGtACTGATAcccttatt<br>4R aatgggcagagtgagattctg	58 °C	288
5	5F TGGTTCCTGTtTTAATGCCct<br>5R ATCCGGTTCAGCCATTAGCCt	58 °C	519
6	6F TGGGGGTGGGAGGGTGGa<br>6R CAAGAtTGACAGgcCCAAGgc	58 °C	433
7	7F tgaactcaagtgatccacctg<br>7R GGAaTCCATAGTTGGGTAGAg	58 °C	463
8	8F TTGCATTCTTCCCgtcacccaac<br>8R AGTCTTTGGTGAaACTAgTAAga	58 °C	390
9	9F ACAGCTGCCTCTCCCACAt<br>9R TCACTTCCTGCCTCCATGGt	58 °C	416
10–11	10F GAGAGCCAGGGCAGAGCCTc<br>11R TAAGCTCACaCTGGCCCTGc	58 °C	548
